# Data for the analysis of interactive multibiomarker responses of a marine crustacean to long-term exposure to aquatic contaminants

**DOI:** 10.1016/j.dib.2018.09.055

**Published:** 2018-09-27

**Authors:** Isabel Oliveira Abreu, Catarina Monteiro, A. Cristina S. Rocha, M.A. Reis-Henriques, Catarina Teixeira, Maria Clara Pires Basto, Marta Ferreira, C. Marisa R. Almeida, Luís Oliva-Teles, Laura Guimarães

**Affiliations:** a CIIMAR - Interdisciplinary Centre of Marine and Environmental Research, University of Porto, Terminal de Cruzeiros do Porto de Leixões, Av. General Norton de Matos s/n, 4450-208 Matosinhos, Portugal; b Faculdade de Ciências da Universidade do Porto, Rua do Campo Alegre, s/n, 4169-007 Porto, Portugal; c Instituto de Ciências Biomédicas Abel Salazar, Rua de Jorge Viterbo Ferreira no 228, 4050-313 Porto, Portugal; d MARE-UC, Incubadora de Empresas da Figueira da Foz, Parque Industrial e Empresarial da Figueira da Foz (Laboratório MAREFOZ), Rua das Acácias Lote 40A, 3090-380 Figueira da Foz, Portugal; e School of Marine Studies, Faculty of Science, Technology and Environment, The University of the South Pacific, Laucala Bay Road, Suva, Fiji

## Abstract

The data presented herein relates to the article entitled “Multibiomarker interactions to diagnose and follow-up chronic exposure of a marine crustacean to hazardous and noxious substances (HNS)” (Abreu et al., 2018). Multibiomarker approaches, including molecular, biochemical, physiological and behaviour parameters, are recognised as valuable and cost-effective to employ in integrated chemical and biological effects monitoring of aquatic contamination. Many biomarkers assessed in such programmes share common physiological pathways, showing concomitant or interdependent responses, which can reflect in increased energy costs related to physiological acclimation. Though, routine single biomarker data analysis, and exploratory principal component analysis, limit information obtained from the data collected and their functional interpretation. Ultimately, this influences the type of management actions taken to protect an affected ecosystem. This article presents data employed to develop an analytical approach accounting for multibiomarker interactions. The method was useful to diagnose and follow-up long-term exposure of the marine green crab (*Carcinus maenas*) to Hazardous and Noxious Substances (HNS).

**Specifications table**

**Table:** 

Subject area	*Biology*
More specific subject area	*Environmental Toxicology*
Type of data	*Tables, figures*
How data was acquired	*Biochemical data was acquired by spectrophotometric measurements (BioTek Power Wave 340 spectrophotometer); bioaccumulation was measured by GC–MS using headspace solid phase microextraction (SPME) in a Varian Saturn 2000 mass spectrometer (Walnut Creek, CA) coupled to a Varian 3900 gas chromatograph; a feeding behaviour assessment was done.*
Data format	*Analysed*
Experimental factors	*Crabs were exposed to environmental contaminants over 21 days. At selected exposure periods samples of muscle, thoracic ganglion, digestive gland and gills were snap frozen in liquid nitrogen; they were processed later for biochemical determinations.*
Experimental features	*Crabs were exposed to low and high concentrations of HNS acrylonitrile or aniline for 21 days. A feeding assay was conducted throughout the exposures (0, 6, 13 and 20 days). At different time points (0, 7, 14 and 21 days) tissues were collected for biochemical analysis.*
Data source location	*CIIMAR, Matosinhos, Portugal.*
Data accessibility	*Data is with this article.*
Related research article	IO Abreu, C Monteiro, ACS Rocha, MA Reis-Henriques, C Teixeira, MCP Basto, M Ferreira, CMR Almeida, L Oliva-Teles, L Guimarães. Multibiomarker interactions to diagnose and follow-up chronic exposure of a marine crustacean to hazardous and noxious substances (HNS). Environ. Pollut. (2018) 242:1137–1145.

**Value of the data**

• Integrated compensatory responses of physiological systems towards homeostasis are generally not investigated when it comes to assess exposure/effects of aquatic contaminants.
• Discriminant Function Analysis (DFA), a hypothesis-driven multivariate analysis, is a useful technique to investigate coordinated or interdependent multibiomarker responses.
• DFA can depict temporal patterns of response to low and high exposure concentrations, identify sets of interactive multibiomarker predictors for each contaminant, and provide an integrated response index informing on detrimental effects and adaptation responses.
• Accounting for multibiomarker interactions can bring, otherwise overlooked, information about animal responses to environmental contaminants and their modes-of-action.

## 1 Data

**Fig. 1:**
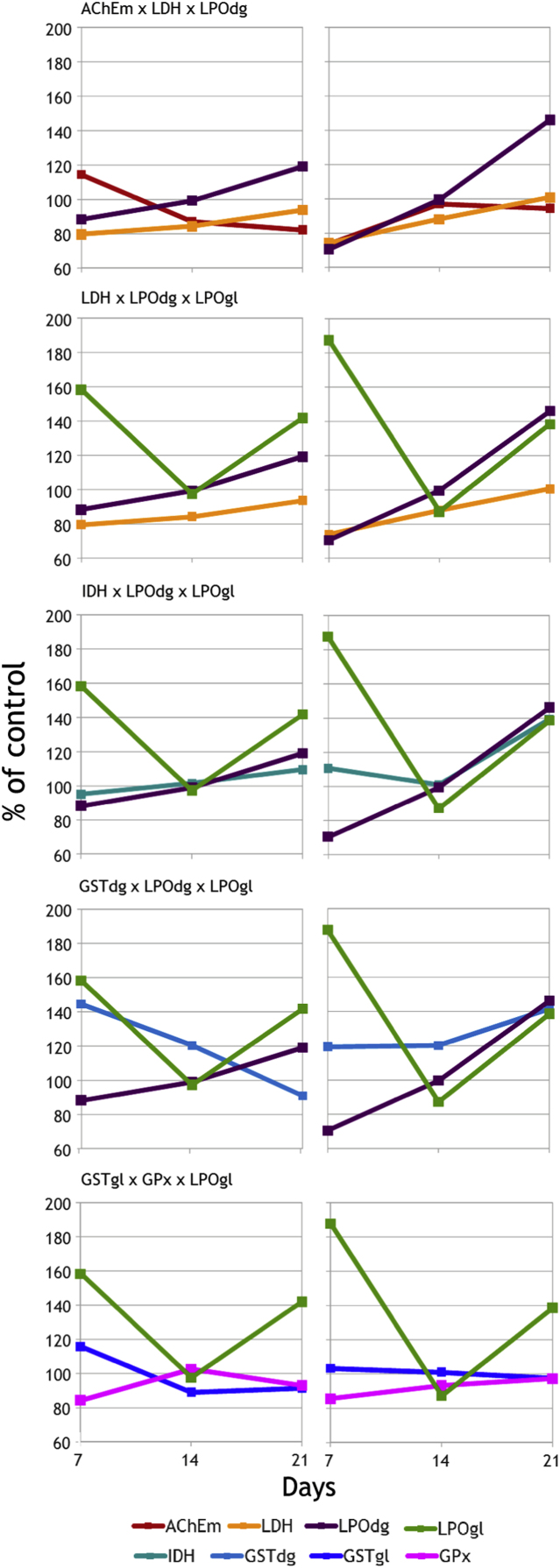
Variation of biomarkers in interactive predictors with higher canonical discriminant coefficients obtained for the lowest (left) and highest (right) concentrations of acrylonitrile tested. *X*-axis represents the duration of exposure in days. AChEm, acetylcholinesterase activity in muscle tissue; LDH, lactate dehydrogenase activity in muscle; IDH, isocitrate dehydrogenase activity in muscle; GSTdg, glutathione *S*-transferases activity in the digestive gland; GPx, glutathione peroxidase activity in the digestive gland; LPOdg, lipid peroxidation in the digestive gland; GSTgl, glutathione *S*-transferases activity in the gills; LPOgl, lipid peroxidation in the gills.

**Fig. 2:**
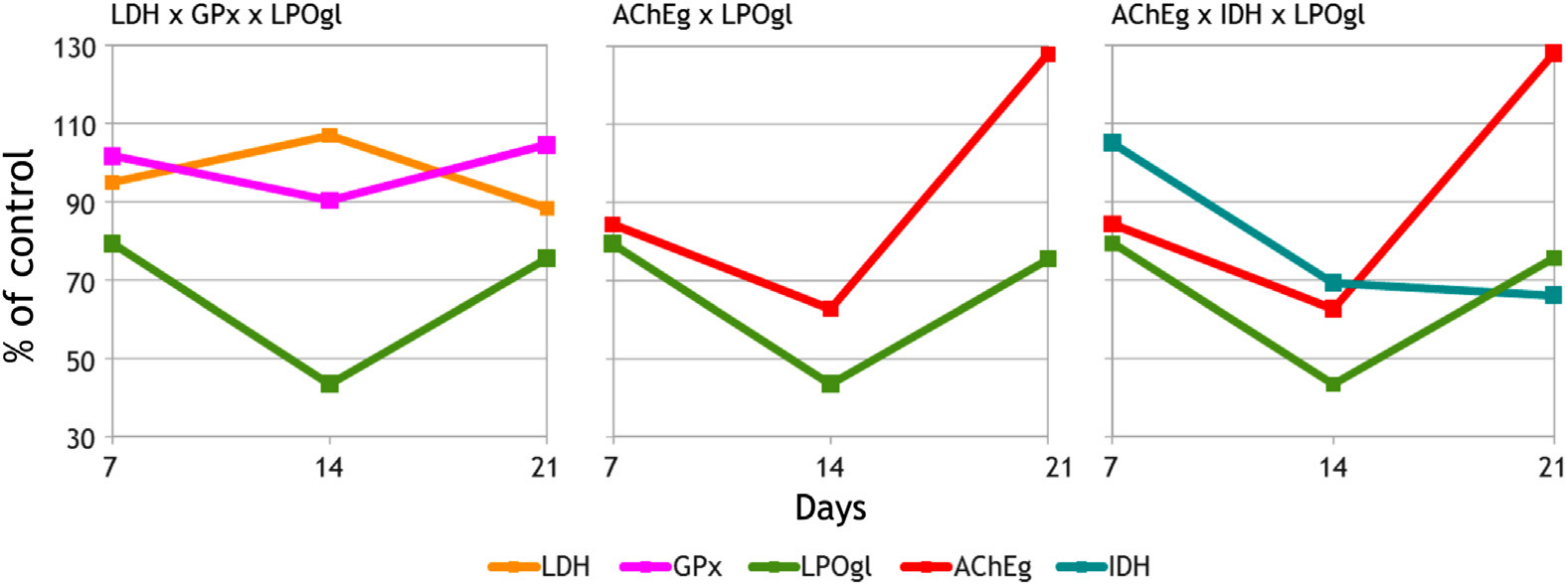
Variation of biomarkers in interactive predictors with higher canonical discriminant coefficients obtained for the highest concentration of aniline tested. *X*-axis represents the duration of exposure in days. AChEg, acetylcholinesterase activity in thoracic ganglion; LDH, lactate dehydrogenase activity in muscle; IDH, isocitrate dehydrogenase activity in muscle; GPx, glutathione peroxidase activity in the digestive gland; LPOgl, lipid peroxidation in the gills.

This data article presents tables showing the results of univariate analysis of feeding behaviours and biochemical determinations in the muscle, thoracic ganglion, digestive gland and gills of *Carcinus maenas* exposed to low and high post-spill concentrations of acrylonitrile or aniline for 21 days ( Tables 1, 2 and 3). Models, and sets of interactive predictors, obtained through Discriminant Function Analysis are shown in Tables 4 and 5. Variation of biomarkers in each final interactive predictor retained in the model are presented for acrylonitrile (Fig. 1) and aniline (Fig. 2). The data provides detailed support to the application of the analytical approach accounting for multibiomarker interactions employed in Abreu et al. [1] to other biomarker datasets, with the aim of diagnosing and follow-up exposure to environmental contamination. This integrated data analysis can be applied to laboratory or field (e.g. Integrated monitoring) studies. Its results can contribute to refine risk estimations for toxicant exposure and impact the type of management actions to be implemented on affected ecosystems.

**Table 1:** Results of full-factorial two-way ANOVAs performed to assess effects of acrylonitrile or aniline concentrations and duration of exposure on *C. maenas*.

Parameter	Source of variation	***Acrylonitrile***			***Aniline***		
		df	*F*	*P*	df	*F*	*P*
Food intake	Treatment	2, 18	4.836	**0.021**	2, 18	0.075	0.928
	Time	2, 18	6.497	**0.008**	2, 18	2.925	0.079
	Treatment × Time	4, 18	0.561	0.694	4, 18	3.391	**0.031**

**Table 2:** Results of full-factorial two-way ANOVAs performed to assess effects of acrylonitrile or aniline concentrations and duration of exposure on neurotransmission and energy production. Acetylcholinesterase activity was determined in the thoracic ganglion (AChEg) and muscle (AChEm). Activity of lactate (LDH) and isocitrate (IDH) dehydrogenases were determined in muscle tissue.

Parameter	Source of variation	**Acrylonitrile**			**Aniline**		
		df	*F*	*P*	df	*F*	P
*Neurotransmission*							
AChEg	Treatment	2, 18	1.029	0.377	2, 18	3.542	0.050
	Time	2, 18	0.238	0.790	2, 18	1.604	0.229
	Treatment × Time	4, 18	1.365	0.285	4, 18	4.433	**0.011**
AChEm	Treatment	2, 18	1.119	0.348	2, 18	0.605	0.557
	Time	2, 18	1.159	0.336	2, 18	1.603	0.229
	Treatment × Time	4, 18	2.817	0.056	4, 18	1.166	0.359
*Energy metabolism*							
LDH	Treatment	2, 18	5.424	**0.014**	2, 18	1.652	0.219
	Time	2, 18	4.737	**0.022**	2, 18	3.217	0.064
	Treatment × Time	4,18	1.132	0.373	4, 18	4.221	**0.014**
IDH	Treatment	2, 18	0.831	0.452	2, 18	2.811	0.087
	Time	2, 18	1.428	0.266	2, 18	20.735	**0.000**
	Treatment × Time	4, 18	1.142	0.369	4, 18	0.625	0.650

**Table 3:** Results of full-factorial ANOVAs performed to assess effects of acrylonitrile or aniline and duration of exposure on biotransformation, anti-oxidant defences and oxidative damage. Activity of glutathione *S*-transferases (GSTdg), glutathione peroxidase (GPx) and levels of lipid peroxidation (LPOdg) were determined in the digestive gland. Activity of glutathione *S*-transferases (GSTgl) and levels of lipid peroxidation (LPOgl) were determined in gills.

Parameter	Source of variation	**Acrylonitrile**			**Aniline**		
		df	*F*	*P*	df	*F*	*P*
*Biotransformation and anti-oxidant defences*							
GSTdg	Treatment	2, 18	4.577	**0.025**	2, 18	0.635	0.542
	Time	2, 18	1.843	0.187	2, 18	0.095	0.910
	Treatment × Time	4, 18	4.858	**0.008**	4, 18	0.300	0.874
GSTgl	Treatment	2, 18	0.096	0.909	2, 18	0.005	0.995
	Time	2, 18	4.188	**0.032**	2, 18	0.595	0.562
	Treatment × Time	4, 18	1.681	0.198	4, 18	0.816	0.532
GPx	Treatment	2, 18	3.453	0.054	2, 18	1.870	0.183
	Time	2, 18	3.803	**0.042**	2, 18	5.387	**0.015**
	Treatment × Time	4, 18	5.028	**0.007**	4, 18	0.345	0.844
*Oxidative damage*							
LPOdg	Treatment	2, 18	0.112	0.894	2, 18	1.675	0.215
	Time	2, 18	7.146	**0.005**	2, 18	0.402	0.675
	Treatment × Time	4, 18	1.111	0.382	4, 18	0.706	0.598
LPOgl	Treatment	2, 18	2.531	0.107	2, 18	2.711	0.094
	Time	2, 18	3.648	**0.047**	2, 18	6.238	**0.009**
	Treatment × Time	4, 18	1.484	0.249	4, 18	0.351	0.840

**Table 4:** Results of the discriminant function analyses performed for the two hazardous and noxious substances (HNS) investigated; chi-square tests with all significant roots and cross-validation (*p* to enter was set to 0.05).

HNS	Significant roots	Eigen value	Canonical R	Wilk׳s Lambda	Chi-square	df	*p*-Value	Significant regressors^a^	Cross-validation (%)^b^	
									Analysis samples	Validation samples
Acrylonitrile	4	379.7	0.9987	0.000025	158.7	76	<0.0001	21	100	85
Aniline	2	1.1	0.7196	0.378525	22.3	12	0.0338	5	77	52
Aniline’^c^	2	1.7	0.7922	0.372470	14.8	4	0.0051	6	95	93

a Number of significant regressors (*p *< 0.05) in each model.

b Percent of correct diagnostics.

c Grouping variable without the lowest test concentration.

**Table 5:** Interactive predictors retained in the final classification models, their standardized canonical discriminant coefficients and cumulative percentage of explained variance accounted for by each function. Biomarkers are: acetylcholinesterase in the ganglion (AChEg) and muscle (AChEm), lactate dehydrogenase (LDH), isocitrate dehydrogenase (IDH), glutathione *S*-transferases in the digestive gland (GSTdg) and gills (GSTgl), glutathione peroxidase in the digestive gland (GPx), lipid peroxidation in the digestive gland (LPOdg) and gills (LPOgl).

**Interactive predictor**	**Function 1**	**Function 2**	***F***_**(6,3)**_	***P***
***Acrylonitrile***				
AChEg x GPx x LPOdg	344	138	4243	<0.0001
AChEg x IDH x GSTdg	177	47	1347	<0.0001
GSTgl x GPx x LPOgl	161	−65	6028	<0.0001
AChEm x LDH	93	24	682	<0.0001
AChEm x GSTdg x GSTgl	72	16	1528	<0.0001
LDH x LPOdg x LPOgl	70	156	3190	<0.0001
AChEg x IDH x LPOgl	51	−29	304	<0.001
AChEm x LDH x LPOgl	25	−12	36	0.007
AChEg x GSTgl x GPx	14	25	121	0.001
AChEg x IDH x GPx	12	−19	38	0.006
LDH x IDH x GSTgl	8	5	14	0.027
AChEg x GSTdg x GPx	−3	−10	10	0.044
LDH x GSTdg x GSTgl	−12	−45	438	<0.001
AChEm x LDH x GSTgl	−36	−25	398	<0.001
GSTgl x LPOdg x LPOgl	−41	114	431	<0.001
AChEm x GSTgl x LPOdg	−71	−156	2521	<0.0001
LDH x GSTdg	−96	−53	3843	<0.0001
GSTgl x GPx x LPOdg	−98	48	346	<0.001
IDH x LPOdg x LPOgl	−228	−8	4516	<0.0001
GSTdg x LPOdg x LPOgl	−296	99	7187	<0.0001
AChEm x LDH x LPOdg	−416	−145	16,196	<0.0001
Explained variance	94.95	99.98		
***Aniline***				
AChEg x IDH x LPOgl	64	354	15	<0.001
AChEm x GSTgl x GPx	−7	116	4.1	0.032
IDH x GSTdg x LPOdg	−79	161	7	0.006
AChEg x LPOgl	−138	350	20	<0.0001
GSTdg x LPOdg x LPOgl	−171	−184	6.8	0.006
LDH x GPx x LPOgl	−290	3	21	<0.0001
Explained variance	69.05	91.94

## 2 Experimental design, materials, and methods

### 2.1 Test organisms

The test organisms were male intermoult crabs. Their average size was 4.5 ± 0.4 cm carapace width (mean ± SD). The animals were caught in Minho estuary with the help of hand nets [2]. This estuary is considered as low impacted by human activities and related chemical contamination [3], [4], [5]. In the laboratory, crabs were maintained for about 21 days in acclimation at 15 ± 1 PSU salinity, 14 ± 1 °C temperature, with low luminosity and continuous aeration. During acclimation, crabs were fed twice a week with frozen squid. The water was renewed after each feeding.

### 2.2 Experimental design

The test substances acrylonitrile and aniline were obtained from Sigma-Aldrich Chemical (Steinheim, Germany). Exposure concentrations were 100 and 1000 μg/L for acrylonitrile, and 5 and 50 μg/L for aniline. All experiments were conducted under a semi-static test regime. The concentrations were chosen based on maximum admissible concentrations and serious risk concentrations found in the available literature, so as to simulate post-spill levels [6], [7], [8], [9]. Exposure media were prepared by dilution with filtered seawater of stock solutions of acrylonitrile or aniline in ultrapure water. Glass aquaria were used in the exposure experiments. Four crabs were placed in each aquaria. The exposure volume was 4 L of either filtered seawater (control group) or exposure media. Three replicate aquaria were prepared for each treatment and time point investigated, namely 7, 14 and 21d. Salinity and temperature were maintained as indicated for the acclimation period. The levels of oxygen in the aquaria were around 80%. Before the beginning of the experiments the crabs were acclimated for four days to the test conditions. Test media were renewed every day by replacing 80% of the exposure volume. After the changing all aquaria were tightly covered with a plastic film to avoid HNS losses due to volatilisation. Every week, before and after media renewal, 10 mL of experimental water from each treatment were collected into dark flasks and frozen at −20 °C for chemical analysis. At 7, 14 and 21d of exposure three replicates of each treatment were dismantled. Animals were weighed and measured and ice-anaesthetised for sample collection. Another, 12 crabs from the acclimation tank were used to evaluate biomarker levels at time zero of the experiment. The tissues collected were the digestive gland, thoracic ganglion, muscle and gills. These samples were immediately snap frozen in liquid nitrogen and stored at -80°C. They were used for analysis of the biochemical markers. Soft tissues remaining were pooled and frozen at −20 °C. These tissues were used in the chemical measurement of acrylonitrile and aniline to assess tissue accumulation levels.

### 2.3 Chemical analysis

Quantification of acrylonitrile or aniline was done in the test media and crab tissues. For tissue analyses, pools of whole soft tissues from several individuals within the same treatment were used to make five replicate measurements. These samples were homogenised with an Ultra-turrax blender (Ika). Quantification of acrylonitrile and aniline was done through headspace solid phase microextraction (SPME); an autosampler CombiPal model (CTC Analytics) with a polydimethylsiloxane-divinylbenzene (PDMS-DVB, polar) fiber from Supelco was used. Analyses were carried out with a mass spectrometer (Varian Saturn 2000, Walnut Creek, CA) coupled to a gas chromatograph (Varian 3900), which was equipped with a split/splitless injector port, a SPME liner (0.75 mm ID), a microseal septum system (Merlin, Half Moon Bay, CA) and a VF-5 ms column (60 mm length × 0.25 mm diameter, 0.25 μm film thickness, Agilent). High purity (99.9995%, Air Liquide) Helium was used as carrier gas (1.0 mL/min constant flow). Acrylonitrile and aniline were identified through their retention times and mass spectra. Quantification was done using the total mass of selected ions. Standard solutions of both compounds, freshly prepared, were employed for external calibrations. For acrylonitrile the limits of detection (LODs) were 15 μg/L (water samples) and 20 ng/g wet weight (tissue samples). For aniline, the LODs were 12.5 ng/L (water samples) and 254 pg/g wet weight (tissue samples).

### 2.4 Feeding assay

The feeding assay was carried out at days 0 (T0), 6, 13 and 20. For this a cross-shaped net was introduced in each glass aquarium to create four equivalent areas; each area contained a single animal. Two weighed portions of frozen squid, sized 1 × 1 × 1 cm^3^, were given every 10 min to each crab for a maximum period of 30 minutes. At the end of this period, the uneaten portions were recovered, dried with absorbent paper, and weighed to assess the amount of eaten squid.

### 2.5 Biochemical determinations

Nine biochemical markers indicative of vital physiological functions were determined as follows: i) for neurotoxicity, the activity of acetylcholinesterase enzyme was measured in the thoracic ganglion (AChEg) and muscle tissue (AChEm); ii) to evaluate energy metabolism, the activities of lactate dehydrogenase (LDH) and NADP^+^-dependent isocitrate dehydrogenase (IDH) enzymes were determined in muscle tissue; iii) for biotransformation, anti-oxidant defences and oxidative damage in the digestive gland the activities of glutathione *S*-transferases (GSTdg) and glutathione peroxidase (GPx), and the levels of lipid peroxidation (LPOdg) were measured; iv) for biotransformation, anti-oxidant defences and oxidative damage in the gills, the activity of glutathione *S*-transferases (GSTgl) and the levels of lipid peroxidation (LPOgl) were measured. The methods employed in biomarker determinations were done as previously established for *C. maenas* [10], [11] in a BioTek Power Wave 340 microplate reader.

Determination of AChE was done by following the increase in absorbance at 412 nm caused by the reaction of thiocholine with 5,5′-dithio-bis-2-nitrobenzoate (DTNB) as described by Ellman et al. [12]. LDH activity was assayed through the method of Vassault [13], by measuring the decrease in absorbance at 340 nm due to NADH oxidation originating from the conversion of pyruvate to lactate. IDH activity was assayed by assessing the increase in absorbance at 340 nm caused by the reduction of NADP^+^, which is mediated by IDH, according to the method developed by Ellis and Goldberg [14]. GST and GPx activities, and LPO levels were determined in the post-mitochondrial supernatant. Quantification of GST activity was done using the method described by Habig and colleagues [15], which involves the conjugation of glutathione with 1-chloro-2,4-dinitrobenzene, a colour reaction that can be followed at 340 nm. GPx activity was quantified by measuring the decrease in NADPH at 340 nm while employing hydrogen peroxide as substrate, as indicated in Mohandas et al. [16]. LPO was assayed by quantifying at 535 nm the thiobarbituric acid reactive substances (TBARS) formed after reaction with trichloroacetic acid (TCA), according to Filho et al. [17]. The concentration of protein in samples was quantified using the method of Bradford [18]; bovine γ-globuline was the standard employed.

### 2.6 Data analysis

Aquarium means were considered as statistical units for data analysis. Homogeneity of variances was accepted as indicated by the Levene׳s test. Differences among treatments or exposure periods were investigated using factorial two-way analysis of variance (ANOVA). Discriminant Function Analysis (DFA) was then employed to integrate all data and investigate a possible contribution of multiple biomarker interactions to discriminate test treatments. The dependent variable described all exposure conditions resulting from the combination of the two factors, namely the exposure concentration (low, C1; high, C2) and the exposure period (short, 7d; intermediate, 14d; long, 21d). The control group, representing natural variation in non-exposed crabs, was obtained by pooling all control animals. Two by two and three by three combinations of biomarkers were calculated. These were entered as predictors in the model, together with the single biomarkers, to investigate possible interactive responses triggered by exposure conditions. A cross-validation routine was established to validate the model obtained for each toxicant. In this routine, several model recalculations were performed; in each recalculation three samples, representing 10% of the data were left out of the model [1]. After each recalculation, validation samples (data elements left out) were classified with the respective recalculated model. The performance of the model was determined by employing 162 validation samples. The models were built by forward entry (*p*<0.05) of the predictors. Prior classification probabilities for the categories of the dependent variable, used in case classification, were computed from the data. For each model obtained, a cluster analysis of the significant DFA functions explaining most of the data variance was subsequently used to interpret relationships among the predictors identified. One-way ANOVA followed by the Duncan test was used to determine the homogeneous groups, helping to better identify the contribution of each significant predictor to discriminate the test groups. Statistical analyses were performed in Statistica v13.2.
