# Supplementary material for: Data for the analysis of interactive multibiomarker responses of a marine crustacean to long-term exposure to aquatic contaminants
Source: Data Brief. 2018 Sep 27;21:386–94. doi: 10.1016/j.dib.2018.09.055 (PMC6197765; doi:10.1016/j.dib.2018.09.055)
Supplement: Supplementary file 1 — Supplementary material [file mmc1.doc]

Author Agreement / Declaration and Conflict of Interest

On behalf of me and my coauthors I hereby certify that all authors of this article have seen and approved the final version of the manuscript submitted. They warrant that the article is the authors' original work, has not been the subject of prior publication and is not under consideration for publication elsewhere. The authors declare no existing conflict of interest.

Luís Oliva-Teles

Faculty of Sciences & CIIMAR

University of Porto
